# Integrating Computational and Experimental Methods to Identify Novel Sweet Peptides from Egg and Soy Proteins

**DOI:** 10.3390/ijms25105430

**Published:** 2024-05-16

**Authors:** Jinhao Su, Kaifeng Liu, Huizi Cui, Tianze Shen, Xueqi Fu, Weiwei Han

**Affiliations:** 1 School of Chemical Science and Engineering, Yunnan University, South Outer Ring Road, Chenggong District, Kunming 650000, China; sjh2860251906@163.com (J.S.); 18604948584@163.com (T.S.); 2 Key Laboratory for Molecular Enzymology and Engineering of Ministry of Education, Edmond H. Fischer Signal Transduction Laboratory, School of Life Sciences, Jilin University, 2699 Qianjin Street, Changchun 130012, China; liukf1220@mails.jlu.edu.cn (K.L.); hzcui23@mails.jlu.edu.cn (H.C.); fuxq@jlu.edu.cn (X.F.)

**Keywords:** sweetness, virtual protein hydrolysis, taste prediction, molecular dynamics simulation, electronic tongue

## Abstract

Sweetness in food delivers a delightful sensory experience, underscoring the crucial role of sweeteners in the food industry. However, the widespread use of sweeteners has sparked health concerns. This underscores the importance of developing and screening natural, health-conscious sweeteners. Our study represents a groundbreaking venture into the discovery of such sweeteners derived from egg and soy proteins. Employing virtual hydrolysis as a novel technique, our research entailed a comprehensive screening process that evaluated biological activity, solubility, and toxicity of the derived compounds. We harnessed cutting-edge machine learning methodologies, specifically the latest graph neural network models, for predicting the sweetness of molecules. Subsequent refinements were made through molecular docking screenings and molecular dynamics simulations. This meticulous research approach culminated in the identification of three promising sweet peptides: DCY(Asp-Cys-Tyr), GGR(Gly-Gly-Arg), and IGR(Ile-Gly-Arg). Their binding affinity with T1R2/T1R3 was lower than −15 kcal/mol. Using an electronic tongue, we verified the taste profiles of these peptides, with IGR emerging as the most favorable in terms of taste with a sweetness value of 19.29 and bitterness value of 1.71. This study not only reveals the potential of these natural peptides as healthier alternatives to traditional sweeteners in food applications but also demonstrates the successful synergy of computational predictions and experimental validations in the realm of flavor science.

## 1. Introduction

Sweetness, one of the five basic tastes, provides a pleasurable sensory experience and enhances human satisfaction with food, and sweetening compounds are widely used in the food industry [1]. However, epidemiological studies have shown that excessive sugar intake increases susceptibility to obesity, type 2 diabetes, cardiovascular diseases (CVDs), metabolic syndrome, and dental caries, thereby impacting human health [2,3,4]. Obesity has become a prominent health issue, leading to reduced life expectancy and quality of life. Thus, the excessive consumption of sucrose and other high-calorie sweeteners is associated with numerous health problems.

Faced with growing health concerns related to the consumption of traditional sugars, the search for alternative sweeteners has become increasingly important. Thus, the food industry has been seeking healthier low-calorie sweeteners that do not compromise taste. Non-sugar sweeteners (NSS), also known as non-nutritive sweeteners (NNS), intense sweeteners (IS), or low-calorie sweeteners (LCS), including saccharin, cyclamate, aspartame, sucralose, neotame, acesulfame K, stevia, monk fruit, and advantame, are frequently used as sugar substitutes in food formulations. Although LCS offer high sweetness potency and reduced calories, their taste characteristics differ from sucrose and may produce undesirable aftertastes [5], which could affect consumer acceptance. For example, acesulfame K, stevia, and monk fruit often come with noticeable bitterness and metallic tastes [6]. Sucralose shares similar sweetness characteristics with sucrose without causing any apparent bitterness. Nonetheless, it may produce some undesirable flavors in products of different textures [7]. Moreover, unlike sucrose, sucralose does not degrade quickly. While it reaches its maximum sweetness rapidly, it leaves behind a pleasant, lingering sweet aftertaste [6].

Beyond sensory impacts, the effects of NSS on human health remain controversial. While some studies suggest a potential association between saccharin intake and bladder cancer, a significant association with other types of cancer has not been systematically observed in meta-analyses of case–control studies or prospective cohort studies [8,9]. Besides the controversy over NSS and cancer, the impact of NSS on human metabolism is also increasingly scrutinized. Health concerns related to metabolic effects include increased risk of overweight and obesity due to compensatory increases in food intake, type 2 diabetes, cardiovascular diseases, and metabolic syndrome [10]. NSS may disrupt the gut microbiota, leading to a decrease in gut microbes associated with obesity and insulin resistance [11]. Another concern regarding the safety of aspartame is its potential carcinogenicity. Aspartame has been reported to induce oxidative stress in several tissues, including the liver of rodents, characterized by potential carcinogens [12]. Large prospective cohort studies have further raised concerns about its carcinogenicity, with findings suggesting a positive correlation between aspartame intake and overall cancer risk [13,14].

Sweet peptides, a class of peptides that exhibit natural sweetness derived from food sources or synthesized through amino acid synthesis, have garnered contemporary interest. In recent years, several peptides from natural foods, such as pufferfish [15], mulberry seeds [16], and soy sauce [17], have been reported to elicit sweetness, serving as low-calorie substitutes for sugar in food and beverages. With growing consumer health consciousness, sweet peptides hold significant potential for application in the food industry.

The latest advancements in computational biology and bioinformatics have paved the way for in silico identification and characterization of bioactive peptides, streamlining the discovery process. Virtual hydrolysis, bioactivity prediction, and machine learning-based flavor prediction offer high-throughput, cost-effective alternatives to traditional experimental methods. There have been several successful cases, including umami, bitterness blocking peptides, etc. [18,19,20]. Moreover, computational studies, especially those utilizing machine learning for sweetness research, have been extensively carried out [21,22,23] but have not been applied to the screening of sweet peptides.

In summary, there is an urgent need for sweeteners that are healthier and safer for individuals with diabetes. Sweet peptides from natural sources offer a low glycemic index and do not cause blood sugar spikes, making them a valuable alternative for diabetic individuals and others aiming to maintain stable blood sugar levels. Naturally derived protein hydrolysates are safer than synthetic alternatives because they do not introduce potentially harmful molecules that can arise during artificial synthesis. This extraction process minimizes the risk of contaminants and ensures that the sweet peptides remain safe for consumption. Egg and soy proteins are both abundant and cost-effective. By using these sources, we can produce sweet peptides at scale, ensuring availability and affordability while also utilizing byproducts from the food industry, which enhances sustainability.

In this study, we aimed to identify and validate sweet peptides derived from egg and soy proteins through an integrative approach combining virtual hydrolysis, machine learning predictions, computational screening, and experimental validation. By employing a series of bioinformatics tools and molecular simulation techniques, we screened peptides for promising sweetness characteristics and non-toxicity. Predictions confirmed that these peptides exhibit no issues related to absorption, distribution, metabolism, excretion, or toxicity (ADMET). Specifically, they showed no toxicity concerning hERG blockers, human hepatotoxicity, drug-induced liver injury, Ames toxicity, rat oral acute toxicity, skin sensitization, carcinogenicity, eye corrosion, eye irritation, or respiratory toxicity.

**Figure 1:**
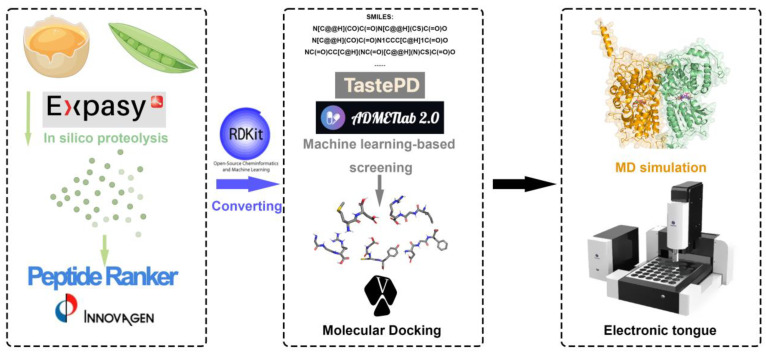
An overview of the process for identifying and validating sweet peptides, including virtual hydrolysis, machine learning-based sweetness prediction, molecular dynamics simulation, and electronic tongue.

The selected peptides were then subjected to testing with an electronic tongue to comprehensively assess their taste properties. Our workflow is illustrated in Figure 1.

This research contributes to the development of food science, especially sweeteners, offering a novel methodology for discovering natural sweeteners. By elucidating the potential of egg and soy protein-derived peptides as sweetening agents, our findings bring hope for the development of healthier food products, aligning with the growing consumer demand for natural and nutritional sugar alternatives.

## 2. Results and Discussion

### 2.1. Identification of Sweet Peptides

**Figure 2:**
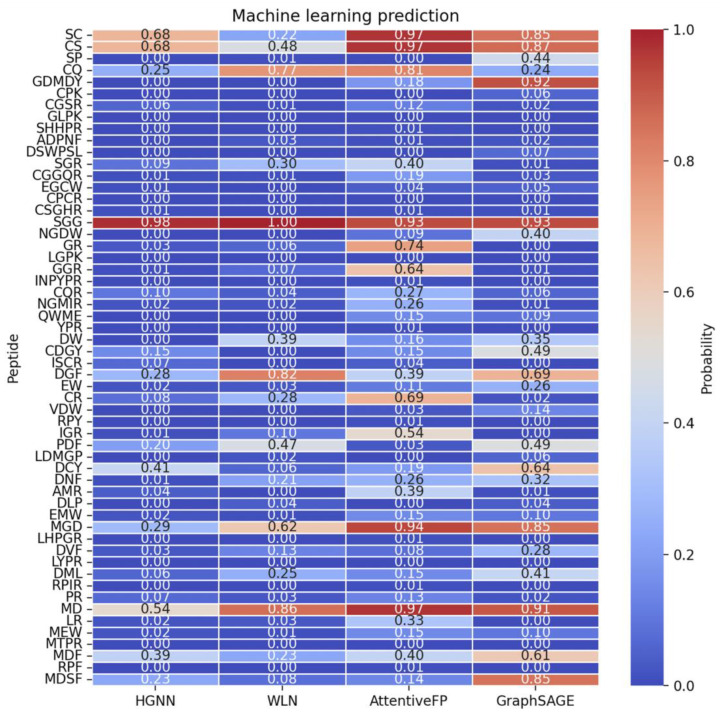
Heatmap of machine learning predictions, displaying the sweetness prediction results for peptides, with values above 0.5 indicating sweetness.

Virtual hydrolysis of proteins was conducted using three typical enzymes: pepsin, trypsin, and chymotrypsin. We chose 2–6 peptides since it is widely known that sweet-tasting molecules are typically small; larger molecules cannot fit into the sweet taste receptor’s binding pocket. This is why both sugars and sweetener molecules are small, whereas larger polysaccharides lack sweetness. This process resulted in the identification of 629 peptides, ranging from 2 to 6 amino acids, derived from soy protein, and 203 peptides from egg protein. Using PeptideRanker (http://distilldeep.ucd.ie/PeptideRanker, 1 January 2024), peptides with a predicted activity greater than 0.5 were considered to have high biological activity. After merging and deduplication, 150 peptides were identified as having high biological activity. These peptides and their respective predicted scores are listed in Table S1. PepCalc was used to calculate solubility, with 56 peptides exhibiting favorable solubility, as detailed in Table S2. Four deep learning methods were employed for sweetness prediction, and the comprehensive results are presented in Table S3. The heatmap of prediction results, as shown in Figure 2, indicates that values above 0.5 are considered sweet. 

WLN predicted 4 peptides, HGNN 5, AttentiveFP 10, and GraphSAGE 10. After merging and deduplication, 15 potential sweet peptides were identified: SC, CS, SGG, MD, CQ, DGF, MGD, GR, GGR, CR, IGR, GDMDY, DCY, MDF, and MDSF. Toxin prediction was conducted to ensure the peptides’ suitability for food consumption, and all potential sweet peptides were found to be non-toxic, as shown in Table 1.

### 2.2. Peptides Screening Based on Structure

The Ramachandran plot validation of the T1R2 and T1R3 structures was in Figure S2. The molecular docking energies of the 15 peptides with T1R2 and T1R3 receptors are displayed in Table 2. Comparing them to aspartame as a control, peptides such as DGF, IGR, GGR, DCY, and MD showed promising binding free energies, suggesting a stronger affinity to the sweet taste receptors and potentially conferring sweetness.

**Figure 3:**
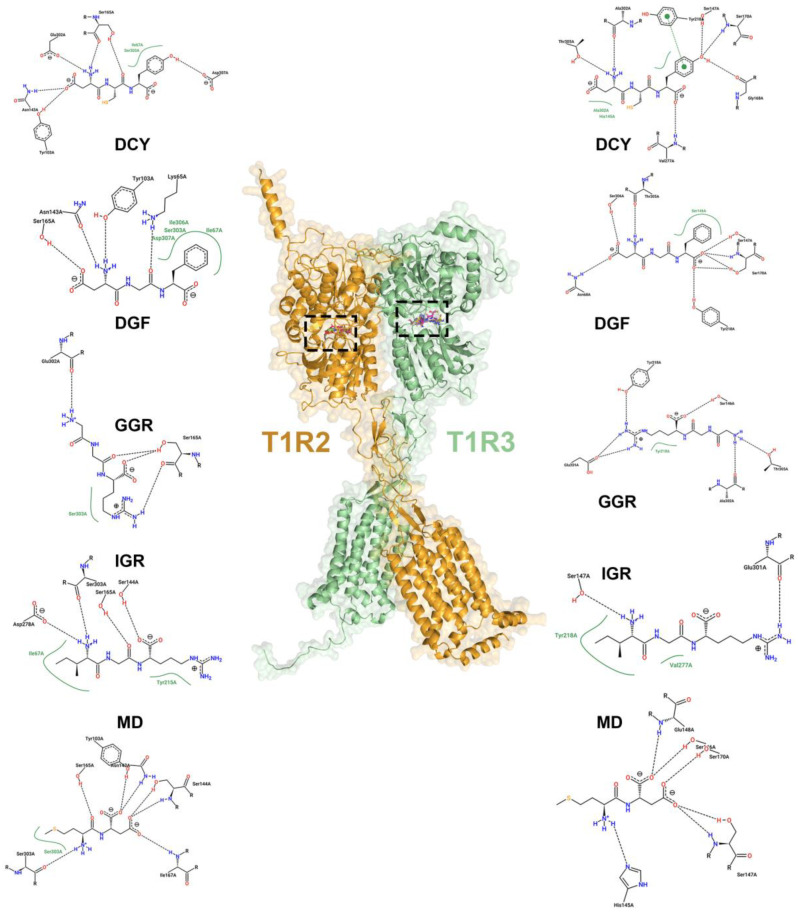
The contact residues and forces between sweet peptides and their binding sites on the protein, the boxes show the active sites.

**Figure 4:**
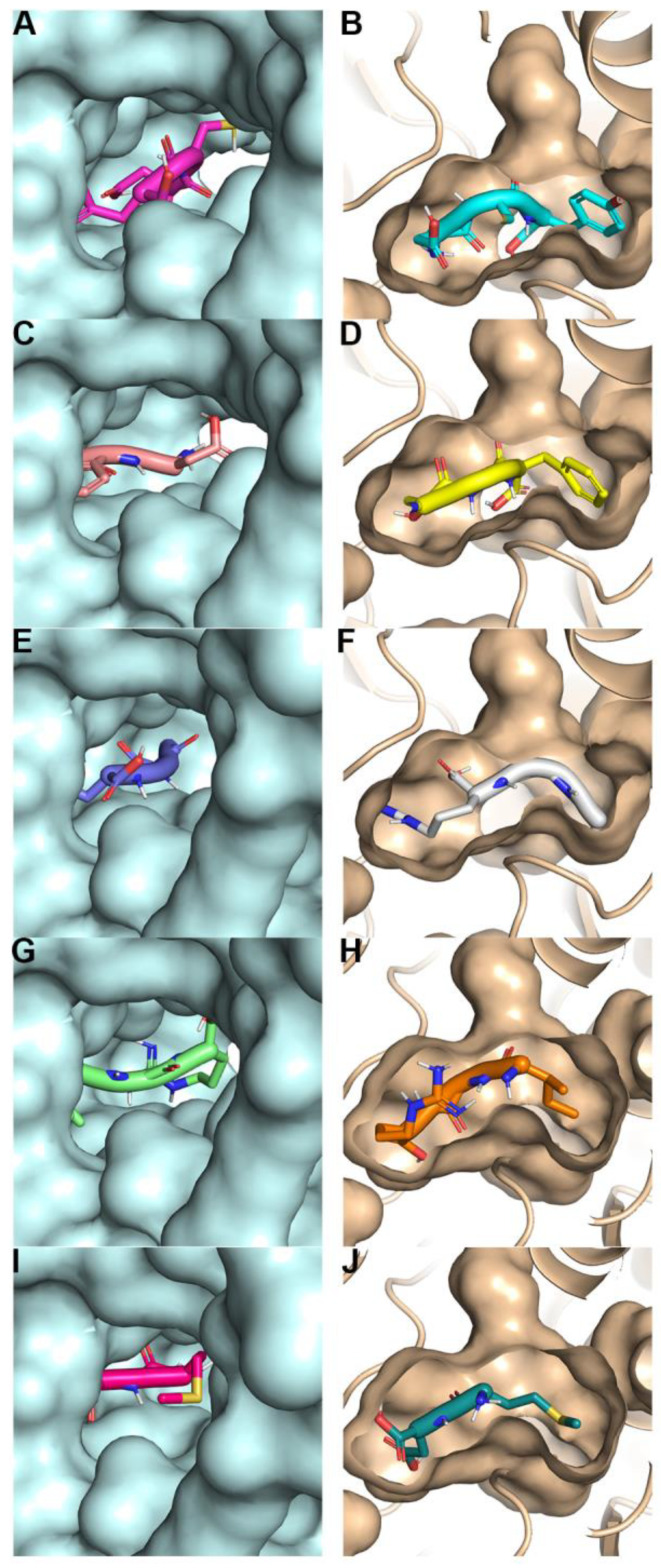
A three-dimensional representation of sweet peptides bound to the sweet taste receptor pocket, highlighting the initial binding conformation. (**A**): T1R2-DCY. (B): T1R3-DCY. (**C**): T1R2-DGF. (**D**): T1R3-DGF. (**E**): T1R2-GGR. (**F**): T1R3-GGR. (**G**): T1R2-IGR. (**H**): T1R3-IGR. (**I**): T1R2-MD. (**J**): T1R3-MD.

These peptides were subjected to 100 ns of molecular dynamics (MD) simulations, and the initial contact residues from the docking are illustrated in Figure 3. The initial 3D conformation of the peptide binding to the protein pocket is shown in Figure 4.

**Figure 5:**
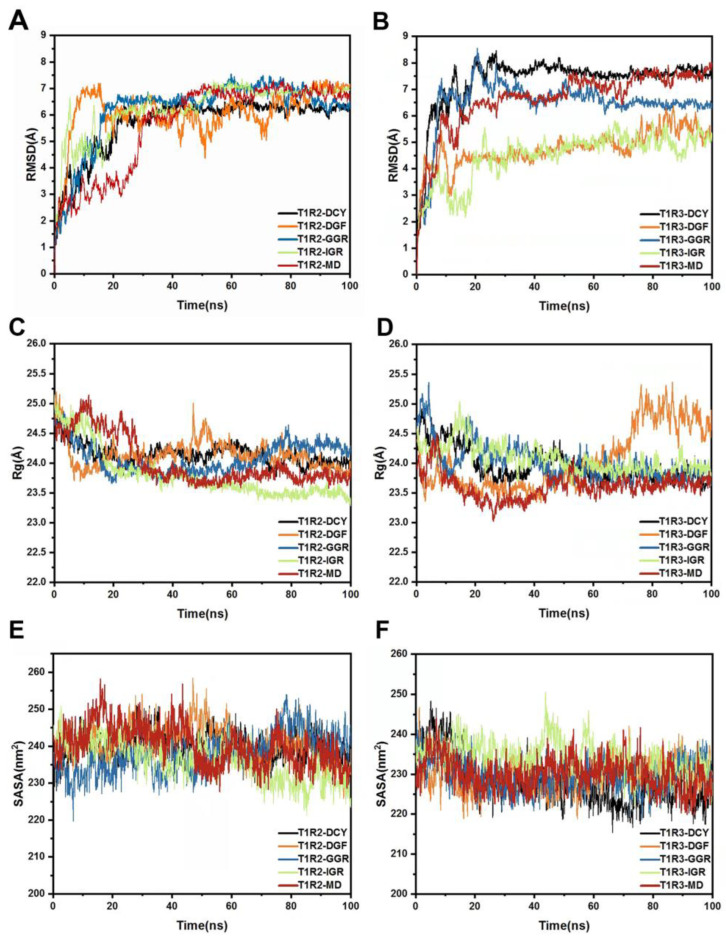
Structural Dynamics of Complex Systems. (**A**): RMSD of the five T1R2 systems. (**B**): RMSD of the five T1R3 systems. (**C**): Rg values of the five T1R2 systems. (**D**): Rg values of the five T1R3 systems. (**E**): SASA of the five T1R2 systems. (**F**): SASA of the five T1R3 systems.

Post-MD simulation, trajectory analysis was conducted. As shown in Figure 5, the Root Mean Square Deviation (RMSD) revealed the deviation of the complex from its initial conformation [24,25]. Fluctuations in RMSD indicated conformational changes during the simulation. T1R2 combined with DGF and MD showed significant RMSD fluctuations, suggesting potential conformational transitions and possibly less stable binding. Similarly, in the T1R3 system, RMSD peaked with DCY and MD, indicating significant conformational changes and potentially less stable binding. The Radius of Gyration (Rg) revealed the compactness of the complex; fluctuations suggest that the binding of peptides to the sweet taste receptors is a dynamic process. Changes in Rg and conformation post-binding altered the binding poses. The Solvent Accessible Surface Area (SASA) indicated that T1R2 combined with IGR and T1R3 with GGR had lower SASA, potentially indicating a tighter binding [26,27].

The MM-PBSA method provided a more accurate prediction of binding energy, as it is based on multiple structure calculations, accounting for dynamic changes during binding. Every 5 ns, one frame was analyzed, totaling 20 frames per trajectory. The results are presented in Table 3. DCY, GGR, and IGR showed higher affinity to T1R2, while GGR, IGR, and DGF showed higher affinity to T1R3. Considering the affinity to both sweet taste receptors, DCY, GGR, and IGR were selected for subsequent validation with the electronic tongue.

### 2.3. Electronic Tongue Analysis

Prior to the electronic tongue experiments, the purity of the three selected peptides was verified using high-performance liquid chromatography–mass spectrometry (HPLC-MS). As illustrated in Figure S1, the purity of each peptide was confirmed to be greater than 95%. Subsequent to this verification, a comprehensive taste assessment of these peptides was conducted using the electronic tongue. The data from three repeated experiments, along with their averages, are presented in Table 4.

In our analysis, the tasteless point, representing the output of the reference solution, served as a benchmark. The reference solution, composed of KCl and tartaric acid, established baseline taste values: −13 for sourness and −6 for saltiness. Thus, if a sample’s taste value fell below the tasteless point, it indicated the absence of that particular taste; conversely, higher values indicated presence.

**Figure 6:**
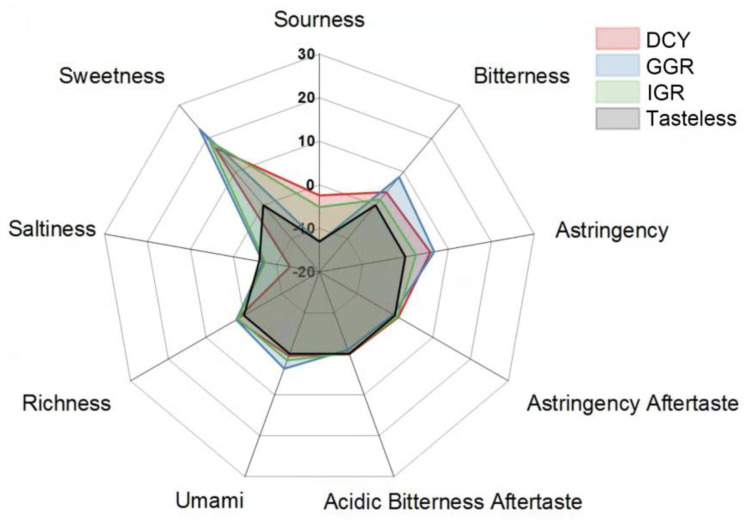
Radar map of the results of the electronic tongue experiment.

To visualize the results more clearly, we employed a radar chart, as shown in Figure 6. The chart revealed that all three peptides exhibited significant sweetness. Notably, DCY and GGR both possessed a slight sourness, whereas IGR did not exhibit any sour taste. Additionally, DCY and IGR showed minor bitter and astringent tastes, which might lead to an unpleasant taste experience. Overall, DCY, IGR, and GGR were characterized as sweet peptides. However, IGR, with its minimal off-tastes and a blend of sweet and sour notes, appears to be more suitable for further application in food products.

Our identification of IGR as the peptide with the most appealing taste profile demonstrates the potential of bioactive peptides in flavor science. Through an innovative approach that integrates advanced computational and experimental techniques, this research provides insights for developing naturally sourced sweeteners. By laying the foundation for healthier alternatives to traditional sweetening agents, it serves as a guide for future studies in flavor science. The discovery of novel sweet peptides like DCY, GGR, and IGR opens up new opportunities for natural sweeteners in the food industry. Their unique taste profiles and high levels of safety make them ideal for low-calorie food and beverage products, offering consumers healthier alternatives to conventional sweeteners. As consumer demand leans toward more health-conscious ingredients, these peptides have significant potential across diverse applications, from baked goods to beverages [28].

Further research should focus on developing efficient extraction and purification methods to enhance the practical utility of these sweet peptides. Testing in various food matrices will reveal the optimal conditions for their integration into commercial products [29]. In vivo studies should confirm their safety and efficacy as sweeteners to meet regulatory standards. This work can be extended by exploring combinations with other natural sweeteners to enhance flavor profiles, stability, and safety in a variety of food applications. Understanding how these peptides interact with other ingredients will help formulate innovative products that meet consumer preferences for health-conscious ingredients. Collaborating with industry stakeholders can accelerate mass production and commercialization while ensuring these naturally sourced sweeteners gain broader adoption in the food industry.

## 3. Materials and Methods

### 3.1. Materials

The sweet peptides were synthesized at Wuhan Dangang Biotechnology Co., Ltd. (Wuhan, China). The purity of peptides was over 95%, and their identities were confirmed by mass spectrometry and high performance liquid chromatography. 

### 3.2. Identification of Sweet Peptides

#### 3.2.1. Virtual Enzymolysis of Protein

Using the sequences of soy protein (NCBI: KRH47534.1) [30], egg envelope protein from Fundulus heteroclitus (GenBank: JAQ51092.1), egg envelope protein EeZPCc from Engraulis encrasicolus (GenBank: ANS71336.1), and another egg envelope protein from Fundulus heteroclitus (GenBank: JAQ89058.1), virtual hydrolysis was conducted. All these sequences are available from the National Center for Biotechnology Information (NCBI), which can be accessed at https://www.ncbi.nlm.nih.gov/ (accessed on 22 December 2023). The ExPASy Peptide Cutter tool (http://web.expasy.org/peptide_cutter/) (accessed on 22 December 2023) [31] was used for the hydrolysis with three typical enzymes: pepsin (pH > 1.3) (EC 3.4.26.1), chymotrypsin-high specificity (C-term to [FYW], not before P) (EC 3.4.21.2), and trypsin (EC 3.4.21.4). The peptides obtained from this hydrolysis were used for subsequent predictions.

#### 3.2.2. Prediction of Biological Activity, Water Solubility

Following virtual hydrolysis, dipeptides to hexapeptides were selected for bioactivity prediction. PeptideRanker (http://distilldeep.ucd.ie/PeptideRanker/) (accessed on 22 December 2023) is a tool used for predicting the potential bioactivity of peptides, with the prediction probability ranging from 0 to 1. Peptides with a score above 0.5 are identified as bioactive [32,33], and peptides with favorable solubility predictions were chosen for further analysis. RDKit was then used for converting peptide sequences to SMILES.

#### 3.2.3. Prediction of Sweetness and Toxicity of Peptides

Subsequently, sweetness prediction was performed using four deep learning methods available on the taste prediction website (https://www.tastepd.com/predict) (accessed on 23 December 2023), which included WLN, HGNN5, AttentiveFP, and GraphSAGE. By taking the union of the results predicted by these methods, we identified a preliminary set of potential sweet peptides. Then, we conducted toxicity screening using ADMET lab 2.0 [34,35,36], retaining peptides predicted to be non-toxic.

### 3.3. Molecular Docking Screening

The amino acid sequences of T1R2 and T1R3 sweet taste receptors were retrieved from the UniProt (T1R2, Primary accession: Q8TE23; T1R3, Primary accession: Q7RTX0). We then constructed the 3D structure of T1R2/T1R3 multimer using Alphafold2 Colab [37,38]. The structure of peptides were generated using openbabel, and molecular docking was performed with Autodock Vina 1.2.0 [39,40]. The size of the docking box was set to x = 60, y = 60 and z = 60, and the spacing between grid points was set to 0.375 Å; ten docking results were generated each, and the conformation of the optimal energy is selected.

### 3.4. Molecular Dynamics Simulation

Systems were designated as follows: T1R2-DCY, T1R2-DGF, T1R2-GGR, T1R2-IGR, T1R2-MD, T1R3-DCY, T1R3-DGF, T1R3-GGR, T1R3-IGR, T1R3-MD. VGF domain of T1R2 (1-490) and T1R3(1-490) were used.

To conduct molecular dynamics simulations on ten different systems, the pmemd.cuda module from AMBER 22 [41] was utilized. For the parameterization of proteins, peptides, and water molecules, the ff14SB [42] and TIP3P [43] force fields in Amber22 were used, the latter of which was also applied to create an octahedral water box around each system. This box was designed with an 8 Å gap from the solute surface, and periodic boundary conditions were established to mitigate edge effects. Sodium ions were added for neutralization.

The hydrogen-containing bonds were constrained through the SHAKE algorithm [44], while the PME method [45] was employed for handling electrostatic interactions, maintaining an 8 Å cutoff. Initial energy minimization, crucial for removing atomic clashes, involved 500 steps each of steepest descent and conjugate gradient algorithms. Subsequently, the systems were gradually heated from 0 K to 300 K in a 50 ps period under the NVT ensemble. The final step involved 100 ns simulations under the NPT ensemble for system equilibration, utilizing a 2 fs timestep and a Langevin thermostat [46] with a 1 ps collision frequency.

### 3.5. Taste Assessment of Peptides Using the Electronic Tongue

In the experimental setup of our study, we employed the SA402B Electronic Tongue from Insent, Atsugi City, Japan, for taste analysis. This device utilizes a sophisticated system of artificial lipid membrane sensors to detect a range of taste sensations. The specific correlations between each sensor and the tastes they detect are detailed in Table 5 of this study.

Our test solutions included a reference solution, consisting of 30 mM potassium chloride and 0.3 mM tartaric acid, which served as the baseline liquid. For cleaning the electrodes, two distinct solutions were prepared: a negative electrode cleaning solution, comprising 100 mM hydrochloric acid mixed with 30% ethanol by volume, and a positive electrode cleaning solution, made up of 10 mM potassium hydroxide, 100 mM potassium chloride, and 30% ethanol by volume.

In preparing the samples, we maintained a solution concentration of 0.1 mg/mL. The protocol for the SA402B Electronic Tongue test involved a multi-step process. Initially, the sensors were cleaned in their respective solutions for 90 s, followed by cleaning in the reference solution for 120 s, repeated twice. The sensor was then zeroed at the equilibrium position for 30 s. During the testing phase, each test lasted for 30 s, with the immediate taste value being output. This was succeeded by a brief 3 s cleaning with the reference solution, after which the sensor was inserted into a new reference solution for an additional 30 s to assess the aftertaste. The taste sensors—C00, AE1, CA0, CT0, AAE, and GL1—were subjected to this procedure four times. The first cycle of each test was discarded, and the average of the last three cycles was computed to obtain the final taste analysis results. This rigorous methodology ensured a reliable and accurate assessment of the taste properties of the samples.

## 4. Conclusions

In this study, we embarked on a comprehensive analysis of peptides derived from egg and soy proteins. Utilizing a virtual hydrolysis approach, we conducted a multifaceted screening process that encompassed assessments of biological activity, solubility, and toxicity. This was complemented by machine learning-based predictions of sweetness and molecular docking screening, and it was further refined through molecular dynamics simulations. The culmination of this rigorous process was the identification of three sweet peptides—DCY, GGR, and IGR—using the electronic tongue for validation. Our findings revealed IGR as the peptide with the most favorable taste profile. This comprehensive approach, integrating advanced computational and experimental techniques, not only underscores the potential of these peptides in food applications but also sets a precedent for future studies in the field of flavor science. The methodologies and insights gained from this research open avenues for the exploration and development of novel, naturally sourced sweeteners, contributing significantly to the food industry’s pursuit of healthier and more palatable alternatives to traditional sweetening agents.

The identification of novel sweet peptides like DCY, GGR, and IGR offers the food industry new possibilities for naturally sourced sweeteners. These peptides can be used in low-calorie food and beverage products, providing consumers with healthier alternatives to traditional sweeteners. Their unique taste profiles, combined with natural origins and high safety levels, make them suitable for a wide range of applications, from baked goods to beverages. This research lays the groundwork for the formulation of innovative, palatable products that align with growing consumer preferences for health-conscious ingredients.

In future research, we recommend further exploration in the following areas: develop more efficient and scalable extraction and purification processes for these sweet peptides to enhance their practical utility; conduct in vivo studies to confirm the safety and efficacy of these peptides as sweeteners, ensuring regulatory compliance and consumer safety; and investigate the stability and taste profile of these peptides in a variety of food matrices, identifying the best conditions for integrating them into food and beverage products. By working closely with industry stakeholders, we will surely address challenges in the mass production and commercialization of these naturally sourced sweeteners.

## Figures and Tables

**Table 1:** Toxicity prediction results of peptides.

Peptide Sequence	SVM Score	Prediction	Hydrophobicity	Steric hindrance	Sidebulk	Hydropathicity	Amphipathicity	Hydrophilicity	Net Hydrogen	Charge	pI	Mol wt
SC	−0.8	Non-Toxin	−0.11	0.57	0.57	0.85	0	−0.35	0.5	0	5.85	208.24
CS	−0.8	Non-Toxin	−0.11	0.57	0.57	0.85	0	−0.35	0.5	0	5.85	208.24
SGG	−0.79	Non-Toxin	0.02	0.63	0.63	−0.53	0	0.1	0.33	0	5.88	219.23
MD	−0.8	Non-Toxin	−0.23	0.77	0.77	−0.8	0	0.85	0.5	−1	3.8	264.31
CQ	−0.8	Non-Toxin	−0.32	0.65	0.65	−0.5	0.62	−0.4	1	0	5.85	249.3
DGF	−0.82	Non-Toxin	0.02	0.71	0.71	−0.37	0	0.17	0.33	−1	3.8	337.36
MGD	−0.81	Non-Toxin	−0.1	0.74	0.74	−0.67	0	0.57	0.33	−1	3.8	321.38
GR	−0.79	Non-Toxin	−0.8	0.68	0.68	−2.45	1.23	1.5	2	1	10.11	231.27
GGR	−0.78	Non-Toxin	−0.48	0.68	0.68	−1.77	0.82	1	1.33	1	10.11	288.34
CR	−0.8	Non-Toxin	−0.86	0.65	0.65	−1	1.23	1	2	1	8.6	277.35
IGR	−0.82	Non-Toxin	−0.29	0.69	0.69	−0.13	0.82	0.4	1.33	1	10.11	344.45
GDMDY	−0.83	Non-Toxin	−0.2	0.74	0.74	−1.36	0	0.48	0.6	−2	3.57	599.67
DCY	−0.74	Non-Toxin	−0.22	0.69	0.69	−0.77	0	−0.1	0.67	−1	3.8	399.44
MDF	−0.84	Non-Toxin	0.05	0.75	0.75	0.4	0	−0.27	0.33	−1	3.8	411.5
MDSF	−0.92	Non-Toxin	−0.03	0.69	0.69	0.1	0	−0.12	0.5	−1	3.8	498.59

**Table 2:** Molecular docking energy of the predicted sweet peptides (kcal/mol).

Peptide	T1R2	T1R3	Sum
DGF	−7.4	−8.2	−15.6
IGR	−7.2	−7.8	−15
GGR	−7.6	−7.1	−14.7
DCY	−6.9	−7.8	−14.7
Aspartame	−7	−7.2	−14.2
MD	−6	−7.8	−13.8
MGD	−6.8	−6.6	−13.4
GR	−6.8	−6.4	−13.2
CR	−6.7	−6.5	−13.2
MDF	−6.9	−6.2	−13.1
CQ	−6.6	−6.4	−13
MDSF	−4.7	−7.9	−12.6
SGG	−6.3	−5.7	−12
SC	−5.6	−6.3	−11.9
GDMDY	−3.8	−8	−11.8
CS	−5.8	−5.6	−11.4

**Table 3:** Results of MM-PBSA (kcal/mol).

Peptide	T1R2	T1R3
DCY	−17.73 ± 2.22	−1.91 ± 3.04
GGR	−16.12 ± 2.17	−34.23 ± 3.90
IGR	−43.07 ± 3.09	−20.86 ± 4.13
DGF	−4.46 ± 2.36	−12.57 ± 2.19
MD	18.82 ± 4.09	−5.16 ± 4.60

**Table 4:** Test results of electronic tongue.

Sample Name	Parallel Experiment Number	Sourness	Bitterness	Astringency	Acidic Bitterness Aftertaste	Astringency Aftertaste	Umami	Richness	Saltiness	Sweetness
Tasteless point		−13	0	0	0	0	0	0	−6	0
DCY	1	−2.33	6.68	7.82	1.06	−0.32	0.32	1.94	−12.96	16.48
	2	−2.49	4.71	6.02	−0.83	−1.47	0.61	1.79	−13.32	17.10
	3	−2.48	0.28	3.74	2.31	2.10	0.60	2.14	−13.20	17.68
	Mean value	−2.43	3.89	5.86	0.85	0.10	0.51	1.96	−13.16	17.09
GGR	1	−13.37	10.65	8.38	0.61	−0.20	3.57	1.85	−6.47	23.15
	2	−13.48	11.58	10.26	−0.74	−1.19	3.77	1.79	−6.94	22.80
	3	−12.73	3.04	1.53	−1.13	−1.46	3.56	2.30	−7.05	22.41
	Mean value	−13.19	8.42	6.72	−0.42	−0.95	3.63	1.98	−6.82	22.79
IGR	1	−4.99	5.73	6.29	1.35	−0.35	1.56	1.47	−7.22	18.73
	2	−5.28	2.19	2.16	0.88	0.46	1.71	1.46	−7.47	19.27
	3	−5.03	−2.79	−0.98	−0.24	−0.77	1.56	1.94	−7.16	19.86
	Mean value	−5.10	1.71	2.49	0.66	−0.22	1.61	1.62	−7.28	19.29

**Table 5:** Matching information between sensors and tastes.

Sensor	Taste	Aftertaste
C00	Bitterness	Acidic Bitterness Aftertaste
AE1	Astringency	Astringency Aftertaste
CA0	Sourness	×
CT0	Saltness	×
AAE	Umami	Richness
GL1	Sweetness	×

## Data Availability

Data is contained within the article and Supplementary Materials.

## Supplementary Materials

The following supporting information can be downloaded at https://www.mdpi.com/article/10.3390/ijms25105430/s1.

## References

[1] Salar F.J., Agulló V., García-Viguera C., Domínguez-Perles R. (2020). Stevia vs. Sucrose: Influence on the Phytochemical Content of a Citrus-Maqui Beverage-A Shelf Life Study. Foods.

[2] Debras C., Chazelas E., Srour B., Kesse-Guyot E., Julia C., Zelek L., Agaësse C., Druesne-Pecollo N., Galan P., Hercberg S. (2020). Total and added sugar intakes, sugar types, and cancer risk: Results from the prospective NutriNet-Santé cohort. Am. J. Clin. Nutr..

[3] Gao M., Jebb S.A., Aveyard P., Ambrosini G.L., Perez-Cornago A., Carter J., Sun X., Piernas C. (2021). Associations between dietary patterns and the incidence of total and fatal cardiovascular disease and all-cause mortality in 116,806 individuals from the UK Biobank: A prospective cohort study. BMC Med..

[4] Moores C.J., Kelly S.A.M., Moynihan P.J. (2022). Systematic Review of the Effect on Caries of Sugars Intake: Ten-Year Update. J. Dent. Res..

[5] Stanner S., Spiro A. (2020). Public health rationale for reducing sugar: Strategies and challenges. Nutr. Bull..

[6] Tan V.W.K., Wee M.S.M., Tomic O., Forde C.G. (2019). Temporal sweetness and side tastes profiles of 16 sweeteners using temporal check-all-that-apply (TCATA). Food Res. Int..

[7] Wagoner T., McCain H., Foegeding E., Drake M. (2018). Food texture and sweetener type modify sweetness perception in whey protein-based model foods. J. Sens. Stud..

[8] Pavanello S., Moretto A., La Vecchia C., Alicandro G. (2023). Non-sugar sweeteners and cancer: Toxicological and epidemiological evidence. Regul. Toxicol. Pharmacol..

[9] Rios-Leyvraz M., Montez J., World Health Organization (2022). Health Effects of the Use of Non-Sugar Sweeteners: A Systematic Review and Meta-Analysis.

[10] Chen L., Wu W., Zhang N., Bak K.H., Zhang Y., Fu Y. (2022). Sugar reduction in beverages: Current trends and new perspectives from sensory and health viewpoints. Food Res. Int..

[11] Turner A., Veysey M., Keely S., Scarlett C.J., Lucock M., Beckett E.L. (2020). Intense Sweeteners, Taste Receptors and the Gut Microbiome: A Metabolic Health Perspective. Int. J. Environ. Res. Public Health.

[12] Riboli E., Beland F.A., Lachenmeier D.W., Marques M.M., Phillips D.H., Schernhammer E., Afghan A., Assunção R., Caderni G., Corton J.C. (2023). Carcinogenicity of aspartame, methyleugenol, and isoeugenol. Lancet Oncol..

[13] Debras C., Chazelas E., Srour B., Druesne-Pecollo N., Esseddik Y., Szabo de Edelenyi F., Agaësse C., De Sa A., Lutchia R., Gigandet S. (2022). Artificial sweeteners and cancer risk: Results from the NutriNet-Santé population-based cohort study. PLoS Med..

[14] McCullough M.L., Hodge R.A., Campbell P.T., Guinter M.A., Patel A.V. (2022). Sugar- and Artificially-Sweetened Beverages and Cancer Mortality in a Large U.S. Prospective Cohort. Cancer Epidemiol. Biomark. Prev..

[15] Zhang M.X., Wang X.C., Liu Y., Xu X.L., Zhou G.H. (2012). Isolation and identification of flavour peptides from Puffer fish (*Takifugu obscurus*) muscle using an electronic tongue and MALDI-TOF/TOF MS/MS. Food Chem..

[16] Bian Y.R., Li W.J., Pan L.H., Peng Q.M., You S., Sheng S., Wang J., Wu F.A. (2022). Sweet-flavored peptides with biological activities from mulberry seed protein treated by multifrequency countercurrent ultrasonic technology. Food Chem..

[17] Zhuang M., Lin L., Zhao M., Dong Y., Sun-Waterhouse D., Chen H., Qiu C., Su G. (2016). Sequence, taste and umami-enhancing effect of the peptides separated from soy sauce. Food Chem..

[18] Zhao W., Zhang S., Wang Y., Ding L., Yu Z. (2023). Identification of bitter receptor T2R14 blocking peptides from egg protein via virtual screening and molecular docking. Food Sci. Anim. Prod..

[19] Song S., Zhuang J., Ma C., Feng T., Yao L., Ho C.T., Sun M. (2023). Identification of novel umami peptides from Boletus edulis and its mechanism via sensory analysis and molecular simulation approaches. Food Chem..

[20] Yu Z., Kang L., Zhao W., Wu S., Ding L., Zheng F., Liu J., Li J. (2021). Identification of novel umami peptides from myosin via homology modeling and molecular docking. Food Chem..

[21] Huang W., Shen Q., Su X., Ji M., Liu X., Chen Y., Lu S., Zhuang H., Zhang J. (2016). BitterX: A tool for understanding bitter taste in humans. Sci. Rep..

[22] Fritz F., Preissner R., Banerjee P. (2021). VirtualTaste: A web server for the prediction of organoleptic properties of chemical compounds. Nucleic Acids Res..

[23] Garg N., Sethupathy A., Tuwani R., NK R., Dokania S., Iyer A., Gupta A., Agrawal S., Singh N., Shukla S. (2017). FlavorDB: A database of flavor molecules. Nucleic Acids Res..

[24] Wang K., Cui H., Liu K., He Q., Fu X., Li W., Han W. (2024). Exploring the anti-gout potential of sunflower receptacles alkaloids: A computational and pharmacological analysis. Comput. Biol. Med..

[25] He Y., Liu K., Cao F., Song R., Liu J., Zhang Y., Li W., Han W. (2024). Using deep learning and molecular dynamics simulations to unravel the regulation mechanism of peptides as noncompetitive inhibitor of xanthine oxidase. Sci. Rep..

[26] Liu K., Guo F., Ma Y., Yu X., Fu X., Li W., Han W. (2023). Functionalized Fullerene Potentially Inhibits SARS-CoV-2 Infection by Modulating Spike Protein Conformational Changes. Int. J. Mol. Sci..

[27] Wang M., Liu K., Ma Y., Han W. (2023). Probing the Mechanisms of Inhibitors Binding to Presenilin Homologue Using Molecular Dynamics Simulations. Molecules.

[28] Nourmohammadi E., Mahoonak A.S. (2018). Health Implications of Bioactive Peptides: A Review. Int. J. Vitam. Nutr. Res..

[29] Zhu Y., Chen G., Diao J., Wang C. (2023). Recent advances in exploring and exploiting soybean functional peptides-a review. Front. Nutr..

[30] Schmutz J., Cannon S.B., Schlueter J., Ma J., Mitros T., Nelson W., Hyten D.L., Song Q., Thelen J.J., Cheng J. (2010). Genome sequence of the palaeopolyploid soybean. Nature.

[31] Gasteiger E., Gattiker A., Hoogland C., Ivanyi I., Appel R.D., Bairoch A. (2003). ExPASy: The proteomics server for in-depth protein knowledge and analysis. Nucleic Acids Res..

[32] Mooney C., Haslam N.J., Pollastri G., Shields D.C. (2012). Towards the improved discovery and design of functional peptides: Common features of diverse classes permit generalized prediction of bioactivity. PLoS ONE.

[33] Lafarga T., O’Connor P., Hayes M. (2015). In silico methods to identify meat-derived prolyl endopeptidase inhibitors. Food Chem..

[34] Xiong G., Wu Z., Yi J., Fu L., Yang Z., Hsieh C., Yin M., Zeng X., Wu C., Lu A. (2021). ADMETlab 2.0: An integrated online platform for accurate and comprehensive predictions of ADMET properties. Nucleic Acids Res..

[35] Jiang D., Lei T., Wang Z., Shen C., Cao D., Hou T. (2020). ADMET evaluation in drug discovery. 20. Prediction of breast cancer resistance protein inhibition through machine learning. J. Cheminformatics.

[36] Dong J., Wang N.-N., Yao Z.-J., Zhang L., Cheng Y., Ouyang D., Lu A.-P., Cao D.-S. (2018). ADMETlab: A platform for systematic ADMET evaluation based on a comprehensively collected ADMET database. J. Cheminformatics.

[37] Mirdita M., Schütze K., Moriwaki Y., Heo L., Ovchinnikov S., Steinegger M. (2022). ColabFold: Making protein folding accessible to all. Nat. Methods.

[38] Jumper J., Evans R., Pritzel A., Green T., Figurnov M., Ronneberger O., Tunyasuvunakool K., Bates R., Žídek A., Potapenko A. (2021). Highly accurate protein structure prediction with AlphaFold. Nature.

[39] Eberhardt J., Santos-Martins D., Tillack A.F., Forli S. (2021). AutoDock Vina 1.2.0: New Docking Methods, Expanded Force Field, and Python Bindings. J. Chem. Inf. Model..

[40] Forli S., Huey R., Pique M.E., Sanner M.F., Goodsell D.S., Olson A.J. (2016). Computational protein-ligand docking and virtual drug screening with the AutoDock suite. Nat. Protoc..

[41] Case D.A., Darden T.A., Cheatham T.E., Simmerling C.L., Roberts B.P. (2010). AMBER 11.

[42] Maier J.A., Martinez C., Kasavajhala K., Wickstrom L., Hauser K.E., Simmerling C. (2015). ff14SB: Improving the Accuracy of Protein Side Chain and Backbone Parameters from ff99SB. J. Chem. Theory Comput..

[43] Jorgensen W.L., Chandrasekhar J., Madura J.D., Impey R.W., Klein M.L. (1983). Comparison of simple potential functions for simulating liquid water. J. Chem. Phys..

[44] Hopkins C.W., Le Grand S., Walker R.C., Roitberg A.E. (2015). Long-Time-Step Molecular Dynamics through Hydrogen Mass Repartitioning. J. Chem. Theory Comput..

[45] Darden T., York D., Pedersen L. (1993). Particle mesh Ewald: An N⋅ log (N) method for Ewald sums in large systems. J. Chem. Phys..

[46] Davidchack R.L., Handel R., Tretyakov M.V. (2009). Langevin thermostat for rigid body dynamics. J. Chem. Phys..

