# Supplementary material for: Integrating Computational and Experimental Methods to Identify Novel Sweet Peptides from Egg and Soy Proteins
Source: Int J Mol Sci. 2024 May 16;25(10):5430. doi: 10.3390/ijms25105430 (PMC11121995; doi:10.3390/ijms25105430)
Supplement: Supplementary file 1 [file ijms-25-05430-s001.zip › ijms-2980826-supplementary.pdf]

Table S1

Peptide	Bioactive
CW	0.99581
MW	0.995312
GF	0.994712
CMW	0.993837
GW	0.993164
PW	0.992911
LF	0.986934
LW	0.985244
LMF	0.979926
RPF	0.977766
PFPY	0.969077
APW	0.966955
CPPSF	0.96497
WMA	0.961852
AGW	0.959298
MDF	0.959246
CPCR	0.949732
IF	0.949173
SF	0.948796
QF	0.946135
IW	0.944175
NF	0.941145
DGF	0.939426
HGF	0.938552
PDF	0.934107
SW	0.93391
DW	0.933025
CG	0.931922
QW	0.928524
GP	0.905487
HPIW	0.896869
ML	0.894564
GG	0.88736
PP	0.886915
LMP	0.886515
WPVL	0.880808
EGCW	0.876556
HPNF	0.875719
CR	0.865233

PGL	0.855192
ADPNF	0.852384
MR	0.849148
GGL	0.838635
CY	0.831032
MDSF	0.829954
EMW	0.82038
SHW	0.816324
VF	0.815398
NGDW	0.815121
TW	0.814447
GGR	0.81127
GL	0.808777
SIF	0.805497
MEW	0.801147
PR	0.787626
LGP	0.786814
DSWPSL	0.785783
DNF	0.775374
GR	0.766288
ISW	0.757343
INPYPR	0.756391
YPR	0.754834
QNCMY	0.75044
INF	0.749347
AM	0.74549
SQF	0.743007
GSM	0.742395
GY	0.741592
PPA	0.738574
SSW	0.731445
GSMGY	0.728079
CDGY	0.719177
RPY	0.716662
HGVF	0.702486
APAP	0.70062
SML	0.696547
CSGHP	0.695588
QWME	0.691206
AMR	0.688815
SSAMM	0.68653

LDMGP	0.684233
SSIW	0.68172
LIGC	0.676497
DML	0.667515
LISW	0.665913
AANW	0.66488
NPG	0.664548
LYPR	0.663945
CH	0.660567
CI	0.660168
LGL	0.652338
HSILMP	0.649456
DCY	0.64835
CPK	0.648049
MGD	0.644133
IGG	0.642808
AVW	0.63114
CGGT	0.631018
SC	0.620334
LL	0.618551
LKPTW	0.615503
LHM	0.614823
SGG	0.611845
NM	0.604672
CGGQR	0.603286
TIGCAW	0.601279
CSL	0.601262
NGMIR	0.599087
CGSR	0.59867
LHPGR	0.594419
GDMDY	0.589397
CSGHR	0.589257
EW	0.585631
LNMY	0.585134
SGNIPY	0.580003
IVF	0.57343
SVF	0.573302
LAC	0.571138
LR	0.569984
MD	0.569594
HP	0.566647

GLPK	0.561671
DVF	0.551408
DLP	0.549727
VDW	0.548779
CQR	0.546227
AMQL	0.545898
NTW	0.543895
CQ	0.540359
AGY	0.540291
SHHPR	0.538536
PA	0.53447
RPIR	0.532517
IGR	0.532161
NGNC	0.53085
GH	0.530477
HPNL	0.523186
LGPK	0.516678
LY	0.516639
MTPR	0.515171
SP	0.514894
LQPY	0.51421
CS	0.513886
LAP	0.512708
TCIC	0.510885
SGR	0.508729
ISCR	0.504779
SAC	0.503776
ACSY	0.501397
LIPAIP	0.500751

Table S2

pepcalc	Sequence
0	SW
0	SSIW
1	SC
0	SSW
1	CS
0	ACSY
1	SP
0	CY
0	SAC
0	SHW
0	SSAMM
0	GSMGY
0	APW
0	GSM
0	SGNIPY
1	CQ
0	AANW
1	GDMDY
0	SML
0	CG
0	QW
1	CPK
0	HSILMP
0	CSL
0	CSGHP
0	CPPSF
1	CGSR
1	GLPK
0	APAP
0	SQF
0	AMQL
1	SHHPR
1	ADPNF
1	DSWPSL
0	NGNC
1	SGR
1	CGGQR
0	AM
1	EGCW

1	CPCR
0	SF
0	CMW
0	AGW
1	CSGHR
0	GW
1	SGG
0	GP
0	TW
0	CGGT
0	LKPTW
0	AGY
0	NTW
0	NM
1	NGDW
1	GR
1	LGPK
0	GH
1	GGR
1	INPYPR
1	CQR
1	NGMIR
1	QWME
1	YPR
0	SIF
0	GGL
0	CI
1	DW
1	CDGY
0	PW
0	LAC
0	GF
0	GG
0	AVW
1	ISCR
0	PA
0	NPG
0	TIGCAW
0	GL
0	PGL
1	DGF

1	EW
0	LAP
0	TCIC
0	HPNL
1	CR
1	VDW
0	IGG
0	SVF
0	LGP
1	RPY
1	IGR
1	PDF
0	PPA
0	HGVF
1	LDMGP
1	DCY
1	DNF
0	WPVL
0	LIGC
0	ISW
1	AMR
0	LIPAIP
0	HGF
1	DLP
1	EMW
1	MGD
1	LHPGR
0	WMA
1	DVF
0	VF
1	LYPR
0	QNCMY
0	LISW
1	DML
0	NF
0	LGL
1	RPIR
0	GY
1	PR
0	LL
1	MD

0	LW
1	LR
0	IVF
0	ML
0	HPIW
0	PP
0	IW
0	LQPY
0	HPNF
0	LHM
0	LMP
0	CH
1	MEW
1	MTPR
0	IF
1	MDF
0	INF
0	LF
0	RPF
0	HP
0	LMF
1	MDSF
0	LY
0	MR
0	LNMY
0	QF
0	MW
0	PFPY

Table S3

Peptide	SMILES	HGNN			WLN			AttentiveFP			GraphSAGE		
SC	N[C@@H](CO)C(=O)N[C@@H](CS)C(=O)O	Sweet	0.68	0.32	Bitter	0.22	0.78	Sweet	0.97	0.03	Sweet	0.85	0.15
CS	N[C@@H](CS)C(=O)N[C@@H](CO)C(=O)O	Sweet	0.68	0.32	Bitter	0.48	0.52	Sweet	0.97	0.03	Sweet	0.87	0.13
SP	N[C@@H](CO)C(=O)N1CCC[C@H]1C(=O)O	Bitter	0	1	Bitter	0.01	0.99	Bitter	0	1	Bitter	0.44	0.56
CQ	NC(=O)CC[C@H](NC(=O)[C@@H](N)CS)C(=O)O	Bitter	0.25	0.75	Sweet	0.77	0.23	Sweet	0.81	0.19	Bitter	0.24	0.76
GDMDY	CSCC[C@H](NC(=O)[C@H](CC(=O)O)NC(=O)CN)C(=O)N[C@@H](CC(=O)O)C(=O)N[C@@H](Cc1ccc(O)cc1)C(=O)O	Bitter	0	1	Bitter	0	1	Bitter	0.18	0.82	Sweet	0.92	0.08
CPK	NCCCC[C@H](NC(=O)[C@@H]1CCCN1C(=O)[C@@H](N)CS)C(=O)O	Bitter	0	1	Bitter	0	1	Bitter	0	1	Bitter	0.06	0.94
CGSR	N=C(N)NCCC[C@H](NC(=O)[C@H](CO)NC(=O)CNC(=O)[C@@H](N)CS)C(=O)O	Bitter	0.06	0.94	Bitter	0.01	0.99	Bitter	0.12	0.88	Bitter	0.02	0.98
GLP	CC(C)C[C@H](NC(=O)CN)C(=O)N1CCC[C@H]1C(=O)N[C@@H](CCCCN)C(=O)O	Bitt	0	1	Bitt	0	1	Bitter	0	1	Bitter	0	1

K		er			er								
SHHPR	N=C(N)NCCC[C@H](NC(=O)[C@@H]1CCCN1C(=O)[C@H](Cc1c[nH]cn1)NC(=O)[C@H](Cc1c[nH]cn1)NC(=O)[C@@H](N)CO)C(=O)O	Bitter	0	1	Bitter	0	1	Bitter	0.01	0.99	Bitter	0	1
ADPNF	C[C@H](N)C(=O)N[C@@H](CC(=O)O)C(=O)N1CCC[C@H]1C(=O)N[C@@H](CC(N)=O)C(=O)N[C@@H](Cc1ccccc1)C(=O)O	Bitter	0	1	Bitter	0.03	0.97	Bitter	0.01	0.99	Bitter	0.02	0.98
DSWPSL	CC(C)C[C@H](NC(=O)[C@H](CO)NC(=O)[C@@H]1CCCN1C(=O)[C@H](Cc1c[nH]c2ccccc12)NC(=O)[C@H](CO)NC(=O)[C@@H](N)CC(=O)O)C(=O)O	Bitter	0	1	Bitter	0	1	Bitter	0	1	Bitter	0.07	0.93
SGR	N=C(N)NCCC[C@H](NC(=O)CNC(=O)[C@@H](N)CO)C(=O)O	Bitter	0.09	0.91	Bitter	0.3	0.7	Bitter	0.4	0.6	Bitter	0.01	0.99
CGGQR	N=C(N)NCCC[C@H](NC(=O)[C@H](CCC(N)=O)NC(=O)CNC(=O)CNC(=O)[C@@H](N)CS)C(=O)O	Bitter	0.01	0.99	Bitter	0.01	0.99	Bitter	0.19	0.81	Bitter	0.03	0.97
EGCW	N[C@@H](CCC(=O)O)C(=O)NCC(=O)N[C@@H](CS)C(=O)N[C@@H](Cc1c[nH]c2ccccc12)C(=O)O	Bitter	0.01	0.99	Bitter	0	1	Bitter	0.04	0.96	Bitter	0.05	0.95
CPCR	N=C(N)NCCC[C@H](NC(=O)[C@H](CS)NC(=O)[C@@H]1CCCN1C(=O)[C@@H](N)CS)C(=O)O	Bitter	0	1	Bitter	0	1	Bitter	0	1	Bitter	0	1
CSGHR	N=C(N)NCCC[C@H](NC(=O)[C@H](Cc1c[nH]cn1)NC(=O)CNC(=O)[C@H](CO)NC(=O)[C@@H](N)CS)C(=O)O	Bitter	0.01	0.99	Bitter	0	1	Bitter	0.01	0.99	Bitter	0.01	0.99
SG	N[C@@H](CO)C(=O)NCC(=O)NCC(=O)O	Sw	0.	0.	Sw	1	0	Swee	0.	0.	Swee	0.	0.

G		eet	98	02	eet			t	93	07	t	93	07
NGDW	NC(=O)C[C@H](N)C(=O)NCC(=O)N[C@@H](CC(=O)O)C(=O)N[C@@H](Cc1c[nH]c2ccccc12)C(=O)O	Bitter	0	1	Bitter	0	1	Bitter	0.09	0.91	Bitter	0.4	0.6
GR	N=C(N)NCCC[C@H](NC(=O)CN)C(=O)O	Bitter	0.03	0.97	Bitter	0.06	0.94	Sweet	0.74	0.26	Bitter	0	1
LGPK	CC(C)C[C@H](N)C(=O)NCC(=O)N1CCC[C@H]1C(=O)N[C@@H](CCCCN)C(=O)O	Bitter	0	1	Bitter	0	1	Bitter	0	1	Bitter	0	1
GGR	N=C(N)NCCC[C@H](NC(=O)CNC(=O)CN)C(=O)O	Bitter	0.01	0.99	Bitter	0.07	0.93	Sweet	0.64	0.36	Bitter	0.01	0.99
INPYPR	CC[C@H](C)[C@H](N)C(=O)N[C@@H](CC(N)=O)C(=O)N1CCC[C@H]1C(=O)N[C@@H](Cc1ccc(O)cc1)C(=O)N1CCC[C@H]1C(=O)N[C@@H](CCCNC(=N)N)C(=O)O	Bitter	0	1	Bitter	0	1	Bitter	0.01	0.99	Bitter	0	1
CQR	N=C(N)NCCC[C@H](NC(=O)[C@H](CCC(N)=O)NC(=O)[C@@H](N)CS)C(=O)O	Bitter	0.1	0.9	Bitter	0.04	0.96	Bitter	0.27	0.73	Bitter	0.06	0.94
NGMIR	CC[C@H](C)[C@H](NC(=O)[C@H](CCSC)NC(=O)CNC(=O)[C@@H](N)CC(N)=O)C(=O)N[C@@H](CCCNC(=N)N)C(=O)O	Bitter	0.02	0.98	Bitter	0.02	0.98	Bitter	0.26	0.74	Bitter	0.01	0.99
QWME	CSCC[C@H](NC(=O)[C@H](Cc1c[nH]c2ccccc12)NC(=O)[C@@H](N)CCC(N)=O)C(=O)N[C@@H](CCC(=O)O)C(=O)O	Bitter	0	1	Bitter	0	1	Bitter	0.15	0.85	Bitter	0.09	0.91
Y	N=C(N)NCCC[C@H](NC(=O)[C@@H]1CCCN	B	0	1	B	0	1	Bit	0	0	Bit	0	1

PR	1C(=O)[C@@H](N)Cc1ccc(O)cc1)C(=O)O	itter			itter			ter	.01	.99	ter		
DW	N[C@@H](CC(=O)O)C(=O)N[C@@H](Cc1c[nH]c2ccccc12)C(=O)O	Bitter	0	1	Bitter	0.39	0.61	Bitter	0.16	0.84	Bitter	0.35	0.65
CDGY	N[C@@H](CS)C(=O)N[C@@H](CC(=O)O)C(=O)NCC(=O)N[C@@H](Cc1ccc(O)cc1)C(=O)O	Bitter	0.15	0.85	Bitter	0	1	Bitter	0.15	0.85	Bitter	0.49	0.51
ISCR	CC[C@H](C)[C@H](N)C(=O)N[C@@H](CO)C(=O)N[C@@H](CS)C(=O)N[C@@H](CCCNC(=N)N)C(=O)O	Bitter	0.07	0.93	Bitter	0	1	Bitter	0.04	0.96	Bitter	0	1
DGF	N[C@@H](CC(=O)O)C(=O)NCC(=O)N[C@@H](Cc1ccccc1)C(=O)O	Bitter	0.28	0.72	Sweet	0.82	0.18	Bitter	0.39	0.61	Sweet	0.69	0.31
EW	N[C@@H](CCC(=O)O)C(=O)N[C@@H](Cc1c[nH]c2ccccc12)C(=O)O	Bitter	0.02	0.98	Bitter	0.03	0.97	Bitter	0.11	0.89	Bitter	0.26	0.74
CR	N=C(N)NCCC[C@H](NC(=O)[C@@H](N)CS)C(=O)O	Bitter	0.08	0.92	Bitter	0.28	0.72	Sweet	0.69	0.31	Bitter	0.02	0.98
VDW	CC(C)[C@H](N)C(=O)N[C@@H](CC(=O)O)C(=O)N[C@@H](Cc1c[nH]c2ccccc12)C(=O)O	Bitter	0	1	Bitter	0	1	Bitter	0.03	0.97	Bitter	0.14	0.86
RPY	N=C(N)NCCC[C@H](N)C(=O)N1CCC[C@H]1C(=O)N[C@@H](Cc1ccc(O)cc1)C(=O)O	Bitter	0	1	Bitter	0	1	Bitter	0.01	0.99	Bitter	0	1

IGR	CC[C@H](C)[C@H](N)C(=O)NCC(=O)N[C@@H](CCCNC(=N)N)C(=O)O	Bitter	0.01	0.99	Bitter	0.1	0.9	Sweet	0.54	0.46	Bitter	0	1
PDF	O=C(O)C[C@H](NC(=O)[C@@H]1CCCN1)C(=O)N[C@@H](Cc1ccccc1)C(=O)O	Bitter	0.2	0.8	Bitter	0.47	0.53	Bitter	0.03	0.97	Bitter	0.49	0.51
LDMGP	CSCC[C@H](NC(=O)[C@H](CC(=O)O)NC(=O)[C@@H](N)CC(C)C)C(=O)NCC(=O)N1CCC[C@H]1C(=O)O	Bitter	0	1	Bitter	0.02	0.98	Bitter	0	1	Bitter	0.06	0.94
DCY	N[C@@H](CC(=O)O)C(=O)N[C@@H](CS)C(=O)N[C@@H](Cc1ccc(O)cc1)C(=O)O	Bitter	0.41	0.59	Bitter	0.06	0.94	Bitter	0.19	0.81	Sweet	0.64	0.36
DNF	NC(=O)C[C@H](NC(=O)[C@@H](N)CC(=O)O)C(=O)N[C@@H](Cc1ccccc1)C(=O)O	Bitter	0.01	0.99	Bitter	0.21	0.79	Bitter	0.26	0.74	Bitter	0.32	0.68
AMR	CSCC[C@H](NC(=O)[C@H](C)N)C(=O)N[C@@H](CCCNC(=N)N)C(=O)O	Bitter	0.04	0.96	Bitter	0	1	Bitter	0.39	0.61	Bitter	0.01	0.99
DLP	CC(C)C[C@H](NC(=O)[C@@H](N)CC(=O)O)C(=O)N1CCC[C@H]1C(=O)O	Bitter	0	1	Bitter	0.04	0.96	Bitter	0	1	Bitter	0.04	0.96
EMW	CSCC[C@H](NC(=O)[C@@H](N)CCC(=O)O)C(=O)N[C@@H](Cc1c[nH]c2ccccc12)C(=O)O	Bitter	0.02	0.98	Bitter	0.01	0.99	Bitter	0.15	0.85	Bitter	0.1	0.9
MGD	CSCC[C@H](N)C(=O)NCC(=O)N[C@@H](CC(=O)O)C(=O)O	Bitte	0.29	0.71	Swee	0.62	0.38	Sweet	0.94	0.06	Sweet	0.85	0.15

		r			t								
LHPGR	CC(C)C[C@H](N)C(=O)N[C@@H](Cc1c[nH]cn1)C(=O)N1CCC[C@H]1C(=O)NCC(=O)N[C@@H](CCCNC(=N)N)C(=O)O	Bitter	0	1	Bitter	0	1	Bitter	0.01	0.99	Bitter	0	1
DVF	CC(C)[C@H](NC(=O)[C@@H](N)CC(=O)O)C(=O)N[C@@H](Cc1ccccc1)C(=O)O	Bitter	0.03	0.97	Bitter	0.13	0.87	Bitter	0.08	0.92	Bitter	0.28	0.72
LYPR	CC(C)C[C@H](N)C(=O)N[C@@H](Cc1ccc(O)cc1)C(=O)N1CCC[C@H]1C(=O)N[C@@H](CCCNC(=N)N)C(=O)O	Bitter	0	1	Bitter	0	1	Bitter	0	1	Bitter	0	1
DML	CSCC[C@H](NC(=O)[C@@H](N)CC(=O)O)C(=O)N[C@@H](CC(C)C)C(=O)O	Bitter	0.06	0.94	Bitter	0.25	0.75	Bitter	0.15	0.85	Bitter	0.41	0.59
RPIR	CC[C@H](C)[C@H](NC(=O)[C@@H]1CCCN1C(=O)[C@@H](N)CCCNC(=N)N)C(=O)N[C@@H](CCCNC(=N)N)C(=O)O	Bitter	0	1	Bitter	0	1	Bitter	0.01	0.99	Bitter	0	1
PR	N=C(N)NCCC[C@H](NC(=O)[C@@H]1CCCN1)C(=O)O	Bitter	0.07	0.93	Bitter	0.03	0.97	Bitter	0.13	0.87	Bitter	0.02	0.98
MD	CSCC[C@H](N)C(=O)N[C@@H](CC(=O)O)C(=O)O	Sweet	0.54	0.46	Sweet	0.86	0.14	Sweet	0.97	0.03	Sweet	0.91	0.09
LR	CC(C)C[C@H](N)C(=O)N[C@@H](CCCNC(=N)N)C(=O)O	Bitter	0.02	0.98	Bitter	0.03	0.97	Bitter	0.33	0.67	Bitter	0	1
MEW	CSCC[C@H](N)C(=O)N[C@@H](CCC(=O)O)C(=O)N[C@@H](Cc1c[nH]c2ccccc12)C(=O)O	Bitt	0.09	0.9	Bitt	0.09	0.9	Bitter	0.1	0.8	Bitter	0.1	0.9

		er	2	8	er	1	9		5	5			
MTPR	CSCC[C@H](N)C(=O)N[C@H](C(=O)N1CCC[C@H]1C(=O)N[C@@H](CCCNC(=N)N)C(=O)O)[C@@H](C)O	Bitter	0	1	Bitter	0	1	Bitter	0	1	Bitter	0	1
MDF	CSCC[C@H](N)C(=O)N[C@@H](CC(=O)O)C(=O)N[C@@H](Cc1ccccc1)C(=O)O	Bitter	0.39	0.61	Bitter	0.23	0.77	Bitter	0.4	0.6	Sweet	0.61	0.39
RPF	N=C(N)NCCC[C@H](N)C(=O)N1CCC[C@H]1C(=O)N[C@@H](Cc1ccccc1)C(=O)O	Bitter	0	1	Bitter	0	1	Bitter	0.01	0.99	Bitter	0	1
MDSF	CSCC[C@H](N)C(=O)N[C@@H](CC(=O)O)C(=O)N[C@@H](CO)C(=O)N[C@@H](Cc1ccccc1)C(=O)O	Bitter	0.23	0.77	Bitter	0.08	0.92	Bitter	0.14	0.86	Sweet	0.85	0.15

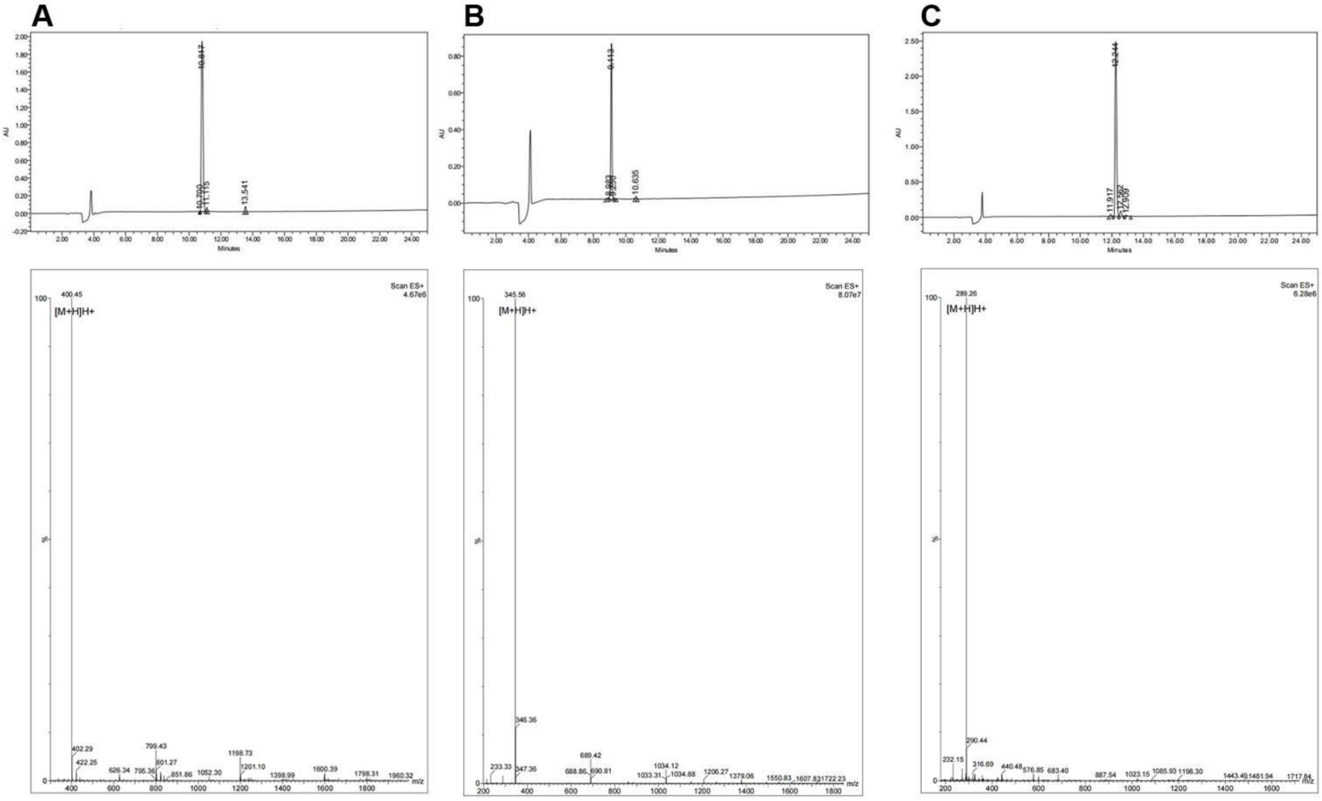

Figure S1 The results of HPLC-MS. A:DCY B: IGR C:GGR.

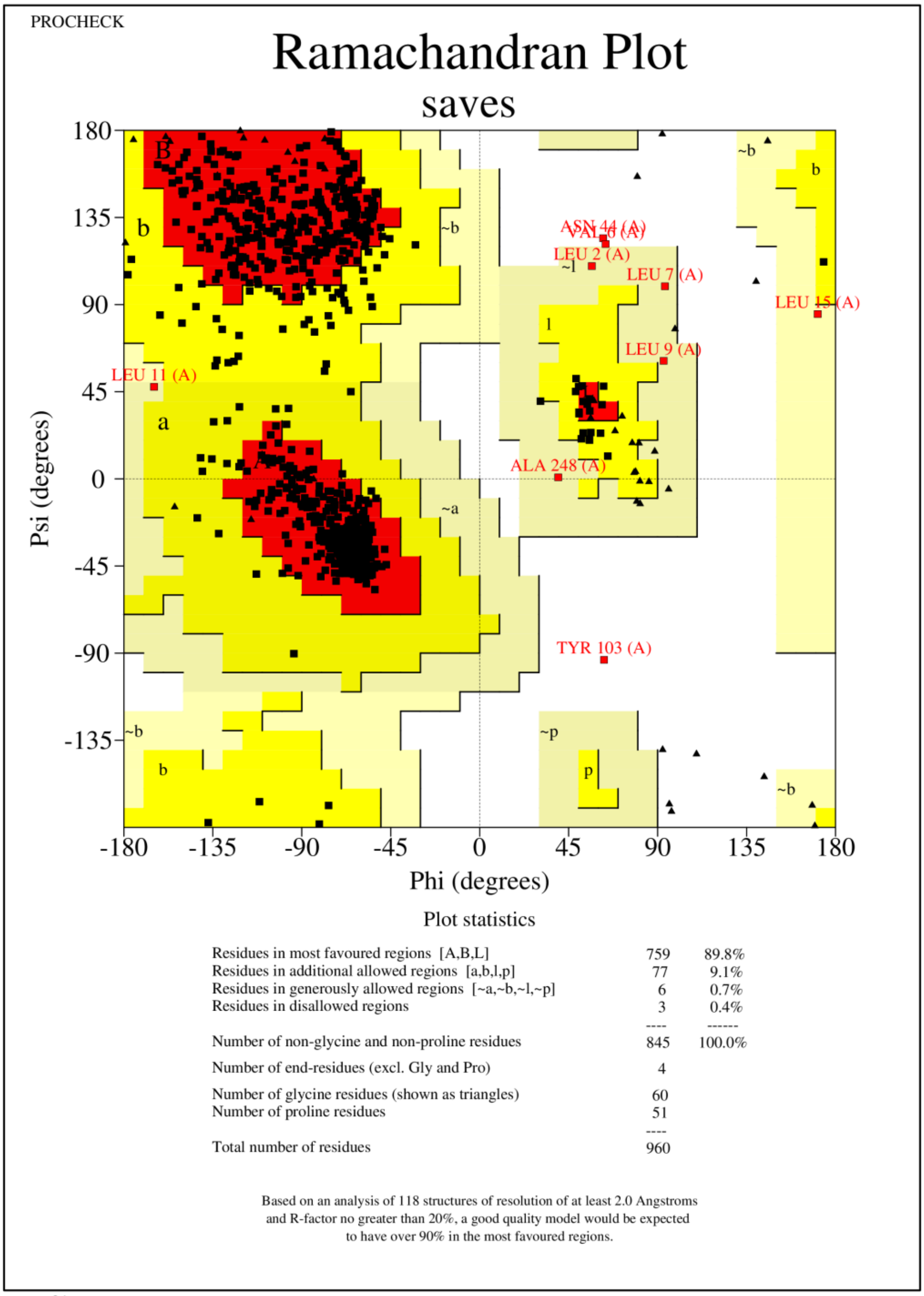

saves_01.ps

Figure S2 The Ramachandran plot validation of the developed structures.
